# Transactions of the Obstetrical Society of Cincinnati

**Published:** 1880-08

**Authors:** 


### Reports of Societies.

### TRANSACTIONS OF THE OBSTETRICAL SOCIETY OF CINCINNATI.

Dr. E. B. Stevens read a paper on The Management of Abortion, upon which the following discussion ensued:

Dr. Goode said there were two things to be kept in view in abortions. The first was the danger from hemorrhage, and the second was from septicæmia. He would be understood as speaking more particularly of cases occurring about the second or third month. If hemorrhage were averted, the danger from septicæmia was very much lessened. During the past few years he had been in the habit of plugging the uterine canal in all severe hemorrhages. For this purpose he had used sponge-tents, prepared to suit each case. There was much opposition to the sponge-tent, but there was one thing to be said in its favor: it was reliable. He would not let the tent remain longer than twelve hours. Nature was competent to take care of cases where there was no hemorrhage. He did not think it an easy matter nor the best plan always to remove the decidua. He did not think much of ergot.

Dr. Palmer objected to the frequent interference in the management of abortions as calculated to be meddlesome and harmful. Many cases did well simply by rest; and masterly inactivity was the key-note of treatment oftentimes.

He approached these cases with much less anxiety than in years past, and believed his then over-anxiety, for fear of death from hemorrhage, led him to interfere unnecessarily.

He did not agree with the statement of Dr. Stevens that ergot only acted on the cervix uteri. Its action was extended over the whole organ; acted best and most on those fibres most largely developed—those of the corpus uteri. So doing, it was useful in restraining hemorrhage, not only by diminishing the size of bleeding vessels, but in expelling the uterine contents. This latter effect is best secured when the cervix was dilated, and the corporeal muscular fibres had unrestricted play.

Of all the agents for controlling hemorrhage, the tampon stood first. He was in the habit of tamponing the vagina in the following way: Place patient on left side, use Sim's speculum, and pack the cervical canal and vaginal vault full of soft muslin. The bladder should be emptied first. If this is done thoroughly, the physician can go about his business or to his rest, and be assured that no great hemorrhage can occur. For this, the most alarming symptom, occurring at end of third month, the tampon was very effectual. Besides, it favored contractions of the body of the uterus, dilatation of the cervix and expulsion of the ovum. In very many instances, after its retention from twelve to twenty-four hours, the ovum will be sufficiently detached and descended to be readily removed from the cervix by the fingers when the tampon is withdrawn; should be changed every twelve hours, and vagina cleansed with carbolized water.

He thought this tampon was safer than sponge tents.

He sometimes employed ice, both externally over the fundus of uterus and into the vagina and cervical canal. It usually acted very promptly and decidedly, and he never saw the least unpleasant symptom, more than the temporary chilling, to arise therefrom. He had injected the uterine cavity with a diluted sulution of persulphate of iron, in years past, for alarming uterine hemorrhages in abortions. While no patient died from effects of such injections, he now considered the practice bad. Temporarily, of course, the flow of blood was checked, but it necessarily filled the uterine cavity with hard, dry clots, irritating to the mucous surfaces, increased materially the chances for septicæmia by a decomposition of these clots, and the danger of phlebitis, metritis, etc.

The instrument which nature has given us is the best to empty the uterus; but he did not approve of attempting this when the ovum was still high and above the internal isthmus. Attempts at removing undetached decidua increase the hemorrhage, as well as every danger incident to abortion. Better wait; tampon and give ergot. He condemed the use of all levers, and hooks, and ovum forceps in these cases.

In chronic hemorrhage, continuing weeks and months after abortion, the cause rested in the retention of some pieces of decidua, etc., and a granular condition of the inner mucous membrane. Here nothing acted so promptly and thoroughly as the curetting of the mucus surface. Usually it is not necessary to dilate the cervix, for it is sufficiently patulous to permit the passage of the curette, a dull-edged copper instrument. If not, he preferred the laminaria tent.

Dr. Thad. A. Reamy said: In teaching, he classed all cases of miscarriage occurring prior to the period of viability as abortions. He presumed, however, that the essayist and authors, whom the essayist criticises, refer to abortions occurring before or about the third month. What he should say, therefore, would be confined to cases occurring thus early. He believed that the estimate of an abortion in ten per cent. of all pregnancies is not too high. He also believed that, excluding criminal abortions, which occur to an extent that is disgraceful to our civilization, a very large proportion of cases is due directly or remotely to syphilitic taint.

When the history of a case, or the symptoms presented to the physician, clearly indicate the existence of this taint, it should be his duty in the interests of humanity, no matter at what period the abortion was threatened, to do nothing to arrest it, but rather facilitate it, since one must recoil at the idea of a syphilitic child being born into the world. Each year's clinical experience added to his conviction that a vast proportion of human ailments have their origin, though often so remotely as not to be appreciated by physicians, much less by communities, in syphilis. And the law which with considerable uniformity guarantees the premature expulsion of the syphilitic ovum should be accepted as a blessing to the race.

Hemorrhage, he stated, is one of the finest signals that an abortion is threatened. The extent to which this hemorrhage is externally manifested does not always represent the degree of certainity that the abortion will be consummated. If it takes place from the lower segment of membranous, or utero-placental attachment, thus securing facility of escape from the uterus so soon as discharged from the vessels, a large amount of blood may be lost without serious interference with the nutrition of the embryo.

He had seen several such cases where the uterus had been excited to such contraction as to dilate the os and even protrude the membranes, and yet, under rest, the sedative action of ergot, or other uterine hæmostatics, the symptoms subsiding and the case going to the normal period of utero gestation, a healthy child being delivered.

When, however, the hemorrhage occurs in the upper field of utero-membranous attachment, the retained blood coagulates, the separation becomes greater and greater as it accumulates, and the disaster is completed. It is proper, therefore, for the practitioner to consider when called to a case of threatened abortion, first—whether it is his duty to interfere with the view of arresting it; secondly—whether interference can be practically successful. Assuming that the abortion must take place, the interests of the embryo now being excluded from all consideration, what is the proper course to pursue? So long as the hemorrhage is not alarming, and there is no evidence of putrescence or septicæmia, nothing special need be done. These complications render abortion dangerous to the mother in the extreme. It could not be denied, however, that in the majority of cases, if the patient could be guided by that intelligence that secures tranquility of mind, and consent to wait patiently for the natural powers to rid the uterus of its contents, this end would be safely accomplished without interference of any character by the physician. At the same time no class of cases more urgently demand professional attention than abortions. First, because of the immediate dangers from hemorrhage and septicæmia; secondly, from the remote results of sub-involution, metritis, endometri[illegible], lymph-angitis, and other evils which might follow even a simple case not properly superintended after the abortion had occurred.

When hemorrhage is profuse, he thought the chief consideration should be to empty the uterus, for this is the only absolutely certain means of permanently arresting the hemorrhage. If, however, the os is not sufficiently dilated to enable the practitioner with facility to reach the ovum and remove it by the fingers, or the fingers aided by a small uterine lever or some such instrument, then the proper method is to tampon, not the vagina, but the cervix. Nothing could be more useless, or, indeed, in some cases more positively injurious, than simply tamponing the vagina under such circumstances, no matter how well done; and it is frequently very imperfectly done.

Such a procedure could only serve to retain the discharged blood with other secretions in contact with the uterus, furnishing opportunity for septic blood lost. For, under such circumstances, if the disposition to hemorrhage be great, the uterus would ascend sufficiently to permit the escape of a dangerous amount of blood above the tampon, even after the most thorough tamponing of the vagina. He thought the method so extensively recommended by modern authors, and eulogized to-night in eloquent words by Dr. Goode, of tamponing the cervix with the sponge-tent, was certainly quite efficient. It controls hemorrhage and promotes dilatation of the cervix. It also promotes uterine contraction and secures ultimately the expulsion of the uterine contents.

Nothing could be more satisfactory than the security with which the physician who has tried this method leaves his patient to meet other calls, or for a night of repose, conscious that she will not suffer seriously from hemorrhage, so long as the tampon remains in situ. And, as a means of double security, the vagina might be tamponed in order to prevent the cervical tampon from escaping from its position, although he had rarely seen any necessity for this precaution.

Notwithstanding what he had just said with reference to the efficiency of the sponge-tent as a tampon, he nevertheless must state frankly that he now never employs this material for such purpose. He would confess that this timidity might have a shadow of superstition it it, but his experience had been such in several instances with the sponge-tent, inducing as it had done, septicæmia of a most violent and dangerous character, that he shrinks from its employment. If the sponge is to remain in but an hour or two, there might be but little danger; but he certainly could not think it safe to allow it to remain eight to twelve hours, as had been recommended by another speaker. We would make this emphatic statement: No uterine cervix can be dilated by the action of the sponge-tent, without abrasion of tissue, the abrasion occurring in a locality liberally supplied with lymphatics.

The idea that air can be excluded under such circumstances is preposterous. The creation of septic material, its retention against the vessels, and its absorption, are, therefore, conditions of great danger. For several years the speaker had been in the habit of tamponing the cervix, in these cases, with shreds of lint or strips of old muslin torn very fine, and crowded into position firmly with a uterine probe, the parts being brought to view by a large sized bivalve speculum.

Now, however, he is in the habit of using absorbent cotton, which answers better than anything else. The quantity of this agent which can be crowded into the cervix is astounding. He saturates it in a one per cent. solution of carbolic acid, squeezing most of the fluid out before inserting. In many instances he substitutes for the carbolic acid a solution of alum, which is at once antiseptic and thorough hæmostatic. The dilating capacity of such a plug, though not quite equal tc sponge, is nevertheless ample in such cases. Such a tampon is more easily expelled than the sponge, but it will not ordinarily be expelled until the dilatation is sufficient to guarantee that the ovum will be thrown down into the cervix, from which it can easily be removed by the fingers, and until it is removed it will at once be a sufficient plug to prevent serious hemorrhage.

With regard to the use of the volsella for dragging the uterus down, in order to facilitate the removal of its contents by the fingers, as recommended by Prof. Simpson in his late contributions to the subject, the speaker stated his objections, not to be from special fear of evil arising from the wounds inflicted in the cervix by the iustruments, for he regarded such wounds as not half so likely to promote septic absorption as those caused by a sponge-tent. But he did object to drawing the uterus too violently downward under such circumstances. He did not fear the influence of this movement upon any of the uterine ligaments so-called, except those posteriorily; but its action upon those he did regard as seriously objectionable.

Moreover, this manipulation and its effect upon the upper portion of the vagina with adjacent structures, he regarded as likely to produce cellulitis. With the uterine contents high up in the cavity, it is frequently quite difficult to secure their removal by the fingers, even when there is sufficient dilatation to admit two fingers without getting the uterus quite low. Indeed, he had found it in some instances more or less difficult when the uterus was quite within reach. He could not understand certain gentlemen who claim that they can always, with the greatest facility, insert the fingers into the uterine cavity and remove the uterine contents at the second or third month of gestation. With the ovum in the lower portion of the uterine cavity and detached, the manipulation is quite easy; and he fancied that if gentlemen who claim to be able so readily to remove it, will measure the length of their fingers and estimate the depth of the uterus at this period, they will consent that probably in most instances they have operated after detachment from the uterine wall and lodgment in the cervix.

In any case where the symptoms demand speedy removal, and he could not otherwise secure this end, he would anæsthetize the patient, pass the hand partly into the vagina, thus enabling him to reach the depth of the uterine cavity with the fingers. He would only do this, however, in cases where he believed the attachment to the uterine wall still exists; in all other cases he had succeeded with the greatest facility in scooping out the ovum and membranes with an instrument shaped much like the open blunt wire curette, but firmer and stronger, which instrument he regards as much more convenient for use and more safe than the placental forceps. With such a lever no injury to the mucous membrane of the uterus is likely to be done.

He considered that far too little attention was usually paid to the after treatment. Other things being equal, the womb and its surroundings do not recover their normal condition in any shorter time after an abortion at three months than after delivery at term. The apparent changes in the gross to be wrought after delivery at term are much greater; but the physiological capacity for these changes is likewise much greater.

It should be the duty of every physician to enforce these points upon the understanding of his patient who has aborted.

In most cases it is judicious to administer ergot once or twice daily for a month after an abortion has occurred. Its action in securing involution is sometimes greatly promoted by the addition of 1-60 gr. of strychnia.

In answer to Dr. Palmer's inquiry why Dr. Reamy objected to the employment of ice bath externally and intra-vaginal for arresting hemorrhages in these cases, Dr. Reamy said: He regarded the employment of ice externally over uterine fundus as safe and often proving efficient in promoting uterine contraction. But he had seen bad effects from crowding ice into the vagina in two cases at least. In one case acute metritis, in the other local peritonitis, which finally became general and destroyed the patient. He was well aware of the almost universal practice of the employment of ice in the vagina; and often it was carried within the uterine cavity, especially when hemorrhage occurs after delivery at a later period of gestation. He was also conscious of the general belief in the total harmlessness of the practice. Nevertheless he was convinced that both theoretical considerations and clinical facts show the practice to be attended with danger.

